# Artificial intelligence for the identification of taphonomic bio-accumulator agents: an actualistic test of potential faunal accumulation agency applied to Tritons Cave (Lleida, Spain)

**DOI:** 10.1098/rsos.241168

**Published:** 2024-10-23

**Authors:** Blanca Jiménez-García, Cristian Micó, Marcos Pizarro-Monzo, Gabriel Cifuentes-Alcobendas, Maite Arilla, Jordi Rosell, Florent Rivals, Ruth Blasco, Enrique Baquedano, Manuel Domínguez-Rodrigo

**Affiliations:** ^1^ Instituto de Evolución en África (IDEA), Universidad de Alcalá, C. Covarrubias 36, Madrid 28010, Spain; ^2^ Departamento de Historia y Filosofía, Facultad de Filosofía y Letras, Área de Prehistoria, Universidad de Alcalá, C. Colegios 2, Alcalá de Henares 28801, Spain; ^3^ Institut Català de Palaeoecologia Humana i Evolució Social (IPHES-CERCA), Zona Educacional 4, Campus Sescelades URV (Edifici W3), Tarragona 43007, Spain; ^4^ Departament d’Història i Història de l’Art, Universitat Rovira i Virgili (URV), Avinguda de Catalunya 35, Tarragona 43002, Spain; ^5^ ICREA, Pg. Lluís Companys 23, Barcelona 08010, Spain; ^6^ Museo Arqueológico y Paleontológico de la Comunidad de Madrid, Plaza de las Bernardas s/n, Alcalá de Henares 28801, Spain; ^7^ Department of Anthropology, Rice University, 6100 Main Street, Houston, TX 77005-1827, USA

**Keywords:** taphonomy, convolutional neural networks, bone surface modifications, carnivores, *Panthera pardus*

## Abstract

Studying bone surface modifications (BSMs) in neotaphonomic research is an important aid to reconstruct agency in the archaeological and palaeontological record. The significance of correctly identifying BSMs has led to extensive debates about adequate methodological and interpretive frameworks to identify taphonomic agents and their bone-modifying patterns in dynamic taphonomic processes; especially those involving inter-agency interactions. Recent analytical innovations in the field have given rise to more updated and less biasing methods, including those rooted in multivariate statistics and artificial intelligence algorithms. Among the latter, convolutional neural networks have demonstrated a substantial potential in precision in the identification and characterization of BSMs. In this study, we present a successful application of these methods to reconstructing taphonomic agency in an archaeological context, specifically in Unit 2 of Tritons Cave (Lleida, Spain). Previous interpretations of this palaeontological layer suggested that it was a natural palimpsest created by carnivores, namely leopards. Through the present research, we objectively tested the original felid-accumulation hypothesis associated with the site’s original taphonomic analysis. Our findings provide additional evidence of the primary leopard agency in the formation of the assemblage and underscores the significant potential of transfer learning for identifying taxon-specific carnivore agency.

## 1. Introduction

Accurately identifying taphonomic bone modifications made by carnivores is crucial for reconstructing the role played by these animals in any taphocoenoseis and for understanding the evolution of theirrelationships with humans in the past. In attempting to distinguish among various carnivoran species, several taphonomic studies have explored different patterns present in modern experimental faunal assemblages generated by different mammalian predators, in order to establish criteria for determining taxon-specific agency (e.g. [1–21]). Although many studies over the years have concentrated on classifying carnivores based on size (large-, medium- or small-sized), diet (strict meat-eating or durophagous) or tooth mark size distributions, these approaches can be problematic when dealing with multiple-patterned or multiple-agency scenarios [1,22–25]. These limitations highlight the need to develop more robust methods that can support taphonomy in its attempt to identify carnivore agency in archaeological and palaeontological records. Such methodological advancements would greatly enhance our understanding of past ecosystems and enable more comprehensive reconstructions of human–animal interactions.

In recent times, more objective approaches based on analytical and statistical analyses, which allow working with multiple variables at the same time, have been tested to improve the process of identification and characterization of bone surface modifications (BSMs) [26–33]. For instance, some studies based on geometric morphometrics, and micro-photogrammetry have successfully achieved a more specific classification of BSMs related to different agents, although they may have encountered some limitations concerning the experimental sample sizes upon which they are based [2,34–37]. One particularly promising approach is the use of machine learning algorithms and, more specifically, convolutional neural networks (CNNs) for automatic image classification. These techniques have demonstrated significant potential by mitigating subjectivity in the study and interpretation of BSMs [29,38–42]. By contrast to traditional approaches that focus on quantifying taphonomic attributes for each mark individually, and studying them in isolation, the advent of artificial intelligence (AI) algorithms has highlighted the importance of considering all significant microscopic attributes of the BSMs collectively, including those that escape the human eye. By comparing and analysing these attributes together, deep learning (DL) enables the establishment of comprehensive approaches to BSM analysis resulting in more accurate classifications [38–40,43–49]. For example, only through these methods cut marks imparted on bones that still retained bulk flesh could be differentiated from those imparted on defleshed bones [43]. Other examples include discerning pristine experimental cut marks from cut marks exposed to short intervals of trampling, which modified some of their most diagnostic microscopic features [50,51], or identifying the intervention of specific carnivores through their respective bidimensional tooth mark shapes [40,44–46].

Despite the fact that DL and computer vision (CV) techniques have proved highly effective in processing, analysing and understanding BSMs, most of the implementations have focused exclusively on experimental sets. The application of these novel techniques and methods to the fossil record is still incipient [47,52]. This study presents an additional application of these methods to the reconstruction of site formation through the analysis of the faunal remains recovered from Unit 2 of Tritons Cave (Lleida, Catalonia), dated to the late Upper Pleistocene. Our objective is to test the main hypothesis, resulting from the interpretation derived from the previous more traditional taphonomic study of the Tritons faunal assemblage, which suggested that the bone accumulation might have been produced mainly by leopards [53]. The application of AI to Tritons aims to provide a more detailed interpretation of the assemblages by assessing the intensity of leopard activities and identifying discrete activities of other potential predators. This paper seeks to demonstrate that AI possesses the potential to serve as a high-resolution technique, complementing traditional descriptive methods and enhancing taphonomy’s ability to offer precise interpretations.

## 2. Tritons Cave

Tritons is a small and secluded cave located in the municipality of Senterada (Lleida, Catalonia) on a cliff on the left bank of the Erinyà Canyon and 100 m above the Flamisell River. The dimensions of the entrance are approximately 2.5 m high and 1.5 m wide. Progressing eastwards, a corridor extends for 19 m, with identical dimensions as the cave entrance, eventually leading to a modest chamber known as the ‘Saleta Final’, spanning roughly 6 m^2^. Within the stratigraphic sequence of the cave, four units have been identified so far: Units 0, 1, 2 and 3. This work focuses exclusively on Unit 2, as a revision of the results of the taphonomic study reported by Micó *et al*. [53].

The radiocarbon dates from the lithostratigraphic Unit 2 frame the faunal and lithic assemblage between 23 940 and 25 860 cal BP at 68.2% probability, within the Marine Isotope Stage (MIS) 2.

The original sample analysed in the 2020 study comprised a total of 2125 remains, with 1354 taxonomically identified, 744 classified by size and 27 remaining unidentified. The Iberian ibex (*Capra pyrenaica*) accounted for the largest representation, with a ‘number of identified specimens’ (NISP) of 1031, constituting 48.2% of the sample. Other small and medium-sized herbivores, such as roe deer (*Capreolus capreolus*) and fallow deer (*Dama dama*), were also present, along with bear remains (*Ursus arctos*) and an individual from the *Panthera* genus, of which the two lower canines and the upper left canine have been identified.

As reported in Micó *et al*. [53] in the same study, carnivore alterations were identified in 168 specimens, representing 7.9% of the total NISP. Within this group, scores are the most abundant (*n* = 150), followed by pits (*n* = 99) and crenulated edges (*n* = 45). Punctures (*n* = 8), furrowing (*n* = 3) and peeling (*n* = 1) were less frequently observed.

BSMs were present in all the identified taxa within the faunal group, except for roe deer (*Capreolus capreolus*) and panthera (*Panthera* sp.). *Capra pyrenaica* exhibited the highest number of modifications (*n* = 167), with 7.86% of the 1031 identified remains showing carnivore-induced bone surface alterations. These BSMs were predominantly found in ribs (20.99%), femora (13.58%), vertebrae (12.34%), phalanges (12.34%), tibiae (11.11%) and humeri (8.6%).

Indications of human activity are very scarce, with cut marks found only on two specimens and thermo-alteration on a single fragment.

Post-depositional processes have significantly affected the faunal remains within the assemblage too, as they have been identified in 96% of the total NISP (*n* = 2042). The most prevalent observed modification is chemical corrosion, which has been documented in 67.39% of the assemblage. Abundant coatings of manganese (53.79%) and calcite (54.64%) are also present, appearing in more than half of the bone fragments. Cracking/fissures (24.96%), root-etching (9.17%), rounding (7.21%), striae of trampling (7%) and polishing (0.4%) also contribute to the overall high degree of post-depositional alterations.

Considering the skeletal profiles of the analysed elements, the presence of carnivore marks and their distribution, the abundance of fresh fractures and the scarcity of anthropogenic modifications, the authors concluded that the remains were introduced intact into the cavity by a primary agent other than humans.

Apart from *U. arctos* and *Panthera* sp., both documented in the site, there are other carnivores present in this area during the Late Pleistocene that could have intervened in the Tritons taphocoenosis, i.e.: cave bear (*Ursus spelaeus*), wolf (*Canis lupus*), hyena (*Crocuta Crocuta spelaea*), cave lion (*Panthera leo spelaea*), leopard (*Panthera pardus*), lynx (*Lynx pardinus*), dhole (*Cuon alpinus*) and fox (*Vulpes vulpes*) [54–62].

According to Micó *et al*. [53], the faunal assemblage resulted from an alternation between the hibernation activities of mainly brown bears and occasionally cave bears, and a predator using the cave as a den or shelter. This predator was capable of carrying primarily whole carcasses of small ungulates (ibex), and occasionally larger animals. This characteristic, along with a well-defined pattern of consumption, initially led to the identification of leopards (*P. pardus*) as the main accumulators in the cave. However, the occasional evidence of peeled and digested elements could also suggest low-intensity intervention by other predators, such as bears, hyenas, canids or humans (see [3–5]).

## 3. Material and methods

### 3.1. Faunal sample used for bone surface modification analysis

**Figure 1.**
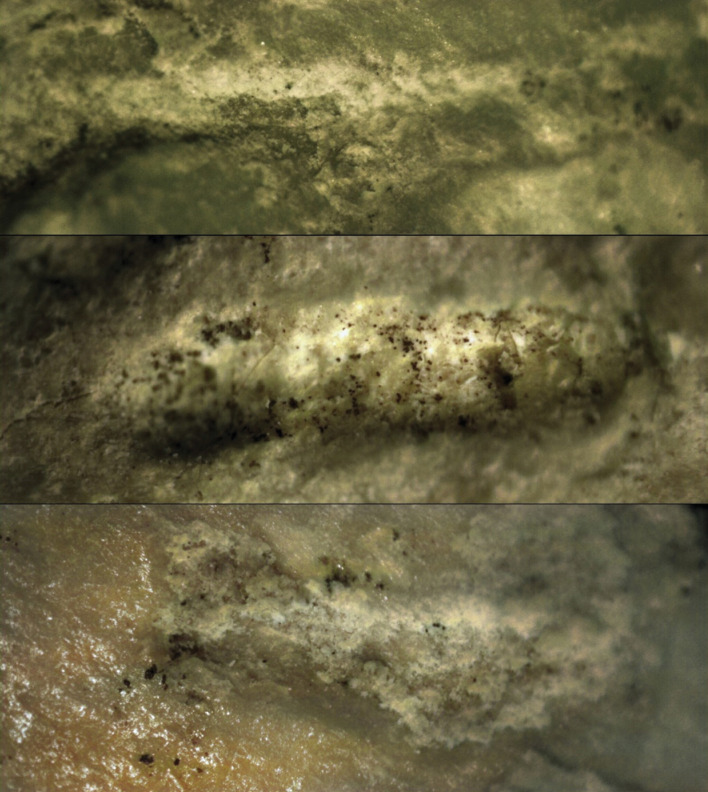
Examples of scores (Tr1, Tr30 and Tr38) documented from Unit 2 of Tritons Cave at 30× magnification.

In the present study, our focus was on tooth marks as described by Blumenschine [63] characterized by ‘u-shaped cross-sections that commonly exhibit noticeable crushing under a hand lens, resulting in a distinct patina compared with the adjacent bone surface.’ We used a sample of 45 two-dimensional images of scores captured with an Optika binocular microscope at 30× magnification to examine the microscopic characteristics of the marks in detail (figure 1). While the original study of Tritons Cave Unit 2 documented approximately 150 potential carnivore scores, we selected only the most unambiguous and the best-preserved ones to mitigate potential biases or identification errors. We discarded those marks that occurred on bones with highly modified bone cortical surfaces, which could mislead the DL analysis of the images.

As for the reference marks used for comparison, we employed modern experimentally derived tooth marks from three different agents to create a graphic library. This sample comprised pits and scores generated by leopards (*n* = 543), wolves (*n* = 200) and hyenas (*n* = 363). We excluded other carnivores such as lions or bears, as they are not known to generate accumulations like the one found in Unit 2 of Tritons Cave and they are not known to intervene on carcasses already defleshed by another primary consumers. Wolves are also not documented to be bone accumulators in caves or open-air dens, but they certainly can impact bones in these locations through post-depositional ravaging, like other durophagous carnivorans. This is the reason why they are included in the present study. Another reasons why lions and bears were excluded are:

- most of the tooth marks documented from Tritons were extracted from dense cortical long bone shafts (both from mid-shafts and metaphyseal portions). Bears virtually leave these anatomical areas damage free, since most of their impact on carcasses is on the axial skeleton, and when damage is inflicted on long bones, it is mostly in the form of furrowing on the cancellous epiphyseal sections [3];
- given the virtually marginal damage inflicted by bears on these anatomical sections, we have not been able to create an image library that would be representative of bear damage on limbs and large enough to be used through AI methods;
- lions are not only not known to accumulate carcasses in caves or dens, but their prey size range is substantially bigger than the ibex [64], which constitutes the bulk of the Tritons faunal assemblage. The only potential open-air accumulation made by lions [65] has recently been re-evaluated as more likely made by leopards and their interaction with hyenas [66];
- the predominance of the small ibex at Tritons indicates a specialized medium-sized predator (which excludes bears and lions);
- given that previous taphonomic work indicated a flesh-eating carcass accumulating agent, we only considered in the present work, bone-accumulating agents (leopards and hyenas) and durophagous carnivores (hyenas and wolves) which could have impacted the assemblage post-depositionally. Other agents not fitting these categories were excluded; and
- lastly, the most conclusive argument to exclude bears and lions from the analysis has a taphonomic nature. Tooth pits documented on the cancellous sections of *Capra pyrenaica* specimens from Tritons are very small on average (*x* = 2.39 length; *x* = 1.87 width on cortical bone, and *x* = 1.62 length; *x* = 1.18 width on dense cortical bone). These mean values are far lower than the mean values (and outside the 95% confidence intervals) of tooth pits documented among bears [6,7,67] and lions [1,6,68]. By contrast, these values for tooth pits are within the dimensions of tooth pits documented in leopards [22,53].

Although claims have been made about the different role of modern and prehistoric wolves as bone accumulators [69], the truth is that no current canid perform carcass accumulation at dens (only foxes have been documented to sometimes bring isolated bones to dens [70]), since all transport food and feed their offspring through regurgitation, and not a single case has been directly documented of wolves accumulating bones at dens at present, nor is there any taphonomically convincing evidence from the past.

The image bank was created using a Leica Emspira 3 microscope, which is capable of capturing both two-dimensional and three-dimensional images. The only exception to this was a part of the leopard tooth mark sample, from the Dar es Salaam collection, which was taken using an Optika binocular microscope and a digital camera (OptiCam3). The Leica Emspira microscope can stack overlapping images of the same mark, eliminating any out-of-focus areas. In contrast to previous studies, we used colour photographs, primarily at 30× magnification because we realized that colour imaging increases microscopic resolution. For very small marks, we enlarged them to ×50 to ensure the details of shape and morphology were visible. These differences were homogenized when images were standardized prior to analysis (see below).

### 3.2. Hyena collection

Three stripped hyenas (*Hyaena hyaena*), all sourced from the Safari Madrid reserve (Aldea del Fresno, Spain), contributed to the creation of the experimental tooth marks sample. The hyenas were an adult female (16 years old) weighing 30 kg, an adult male (18 years old) weighing 35 kg, and an adult female (8 years old) weighing 32 kg. Their regular diet typically consists of 3 kg of lean meat with bone twice a week, supplemented with fruits on the other days. For this particular study, a series of disarticulated and defleshed bones from adult red deer (*Cervus elaphus*) were used, consisting of a total of 27 femora, six humeri, nine radii and 14 tibiae.

Each hyena consumed the bone individually, but they were not allowed to take them to their resting areas shortly after the initial consumption to prevent complete consumption and to allow retrieval of as many bones as possible. The adult male hyena consumed six tibiae, one humerus and seven femora. The adult female hyena consumed nine femora, two humeri, three radii and one tibia. The other adult female hyena modified four femora, two humeri, two radii and one tibia. After each experimental phase, the remains were cleaned by boiling them in water for 6 h and then soaking them in a water and hydrogen peroxide solution for 24 h.

This study sample was complemented by another one, consisting of spotted hyenas (*Crocuta crocuta*), obtained from a den located near Lake Eyasi in Northern Tanzania [67]. All visible and accessible remains were manually collected from the immediate interior of the den, as well as from an approximate 20 m radius surrounding the entrances. With a NISP of 720, the largest portion (84%) of the identified 57 individuals consisted of domesticated animals, including caprines (39%), cattle (24%), domestic dog (12%), donkey (9%) and one pig (2%). Wild taxa specimens included dik-dik (*Madoqua kirkii*), warthog (*Phacochoerus africanus*), Thomson’s gazelle (*Eudorcas thomsonii*), a tragelaphini (Aff. *Tragelaphus scriptus*) and a small primate (Aff. *Galago* sp.). Further details about this sample are given in the original experiment [71].

### 3.3. Leopard collection

The sample of leopard marks, on the other hand, comes from three Persian leopards (*P. pardus saxicolor*), from the Madrid Zoo (Madrid, Spain). The leopard individuals were two 4-year-old males weighing 60 kg, born in captivity, and an adult 9-year-old wild born male weighing 70 kg. The carcasses used in the experiment were tailored to match the leopards’ regular diet, and a total of 12 complete adult sheep limbs (*Ovis aries*) were provided to them. To prevent conflicts, each of the leopards was separately provided with the limbs in anatomical connection.

These experimental procedures were conducted by the zookeepers responsible for the animals, who meticulously documented the process. The young leopards consumed four forelimbs and four hind limbs, while the adult leopard consumed two forelimbs and two hind limbs.

All necessary sanitary protocols were followed during the bone collection process. The recovered remains, as in the case of the hyenas, were boiled and cleaned to remove any remaining meat and fat residues, allowing for detailed documentation of the modifications created.

In addition to the zoo-based experiment, an additional sample of leopard-consumed carcasses was used from a previous study conducted in the Bahari Zoo (Dar es Salaam, Tanzania). In this experiment, a leopard (*P. pardus*) was fed 44 articulated forelimbs (humerus and radius-ulna) and hind limbs (femur and tibia) from 11 goat carcasses (*Capra hircus*) over several weeks. This experiment was first reported by Gidna *et al*. [72]. Tooth marks on these carcasses were observed and photographed with a magnification of 30× using an Optika binocular microscope and a digital camera (OptiCam3). To address depth of field issues, multiple images of each mark were taken at different focus points and rendered using an image enhancer software (Helicon Focus 8.0.4). Pits were documented at various magnifications (10×, 20× and 30×), while scores were documented at a fixed magnification of 30×. Focus stacking techniques were employed to generate images without blurry or out-of-focus areas, ensuring the clarity of the images for further analysis.

### 3.4. Wolf collection

As for the wolf tooth marks sample, it comes from a collection of carcasses belonging to medium and small-sized animals, specifically cervid, ovicaprid and suids, altered by captive Iberian wolves (*Canis lupus signatus*) from the reserve of El Hosquillo (Cuenca, Spain). Five adults were fed disarticulated limb bones (humeri, radii, femora and tibiae) under the surveillance of park keepers and following the protocols and guidelines from the natural reserve.

Bones were exposed for three months and recovered afterwards according to sanitary protocols similar to the ones followed with the previous cited samples. In this case, preparation for analysis and examination was conducted by boiling them in a solution of water and mild soap, with no further chemical additives, and then allowed to air dry [73,74].

### 3.5. Remarks on the experimental collection

The experimental sample intentionally introduced heterogeneity to increase the sampling range of BSM. This is why in the case of hyenas, both fleshed and defleshed bones were used. Although previous studies on experimental generation of cut marks using stone tools clearly showed that there were microscopic differences that could allow the differentiation of cut marks made on fleshed versus defleshed bones [43], a pilot study of tooth marks made on this hyena subsample showed that the tooth marks made by striped hyenas (defleshed bones) and those made by spotted hyenas (a large part on presumably fleshed bones when collected by them) resulted in low discriminatory accuracy and did not show any clear differences. This is probably because tooth enamel is non-abrasive and its impact on bone crushing is similar regardless on whether the tooth impacts bone surfaces with or without covering tissue. By contrast, the abrasive mineral particles of stone tools generate different degrees of microstriations and shoulder flaking when impacting bare bone or tissue-covered bone surfaces.

The sample included wild and captive animals. The stereotypical behaviours of captive animals in zoos and other captivity enclosures has been shown to potentially impact tooth marking intensity and anatomical distribution of damage patterning [8]; however, in the present study, this has no impact, since here we target tooth mark morphology, which depends on the tooth size and shape and not on the biting behaviour. As a matter of fact, the pilot study on the two hyena subsamples (one from wild animals and the other from captive individuals) showed no differences, as we just described above. Experiments were carried out following the official ethical protocols in each of the institutions and countries where they were carried out.

Even though the archeological sample is composed exclusively of scores, we decided to compare them with a referential collection composed of both tooth scores and tooth pits because we realized that the boundaries were frequently hard to establish without measuring the marks and establishing a dimensional ratio (e.g. scores were more than three times longer the width). We also lumped tooth pits and tooth scores together in the present analysis because we showed recently that by increasing image information the models performed better and could differentiate carnivore taxa better because of a more comprehensive dataset [75]. In a previous study using five carnivore taxa, the best performing model achieved an accuracy of 57% [44], whereas in the recent reanalysis of the same carnivores, the mixed tooth set (pits and scores) yielded an accuracy of 80% (worst performing model) and 88% (best performing model) [75].

All the images used for the present study can be found at the public repository: https://doi.org/10.7910/DVN/NHZAIC -https://doi.org/10.7910/DVN/BAJK3I.

### 3.6. Analytical method

When talking about neural networks, we refer to non-linear regression or classification models that mimic the functioning of neurons in living organisms, which are connected to each other and work together [76]; thus, in a neural network, neurons are organized into different layers including an input layer, an output layer and potentially several hidden layers. This is what gives DL its name, since these types of networks can contain up to hundreds of hidden layers.

As for CNNs, they are a specific type of neural network, also composed of a series of neurons with weights, biases and activation functions that can be implemented to learn patterning efficiently, particularly useful in CV tasks, such as image classification [77,78]. In a CNN, neurons receive input in the form of images and use abstraction techniques to analyse and extract relevant information. This information is then processed to generate the output.

In this case, we have resorted to a deep CNN format drawn from transfer learning (TL; i.e. pretrained architectures). We use TL because it deals with models that have been previously trained with thousands of images and a great diversity of morphological elements, thus improving their ability to capture the microscopic features in BSMs. Building on the success of a set of models in previous experimental taphonomic analyses [38,40,43–46,48], we applied the same sequential and residual architectures for the current study, specifically: ResNet 50, VGG19 and DenseNet 201 (version 1.0) [79–82].

The implementation process follows the methodology described in Domínguez-Rodrigo *et al*. [75]. We created individual models for our analysis and then, as a contrasting method, we employed ensemble learning techniques. We used a stacking process with random forest and an extra gradient boosted tree to enhance predictive performance, adjusting hyperparameters with 100 estimators. Given the high but variably accuracy of some of the models, we decided to accept as a confident BSM identification when the three models coincided in the assessment and provided high probabilities. For this purpose, the models used were also used outside the ensemble framework. We split the sample between training (75%) and testing (25%) sets. Images were assigned to each set through randomization. Data augmentation was implemented, through rotation range (40°), width shift range (20%), height shift range (20%), shear range (20%) and horizontal flipping. Images were imported and resized to 250 × 200 pixels. Then we implemented image standardization through the pre-processing function of each of the models used.

In this study, we employed the ‘relu’ and ‘swish’ activation functions in conjunction with the Stochastic gradient descent (SGD) and Adagrad optimizers, with a learning rate of 0.001 and a momentum of 0.9. Additionally, for each of the models, we used a ‘softmax’ activation function in the final fully connected layer, along with categorical cross entropy as the loss function and accuracy as the guiding metric [83]. A dropout rate of 30% was also applied to mitigate overfitting.

Training was performed using size 64 mini-batch cores and testing using size 32 mini-batch cores. The weight update was executed through a 100-epoch backpropagation process. To measure the performance and evaluate the classification results, we relied on accuracy, F_1_ values, and training and loss graphs.

The goal of the present study is to detect the impact of three potential bone accumulators/modifiers that lived sympatrically in the Tritons palaeoenvironment, as is typical of the Upper Pleistocene: hyenas, leopards and wolves. Given the small size of the predominant carcass type accumulated at the site, these three carnivorans seem the most likely candidates as predators and scavengers.

The code used for the present study can also be found at the public repository: https://doi.org/10.7910/DVN/NHZAIC.

## 4. Results

All the three DL architectures (ResNet 50, VGG19 and DenseNet 201) returned accuracy estimates of more than 85% on the testing experimental dataset (table 1). The most successful model was ResNet 50 using Adagrad as the optimizer and relu as the activation function, which yielded almost 92% of accuracy on the testing set and a loss value of 0.23. Results from DenseNet 201, and VGG19 are very similar, all achieving 88% and 85% of accuracy in the classification of the testing set, with slightly higher loss values.

**Table 1.** Details on the performance (accuracy and loss value) of each of the implemented models for the best optimizer–activation function combination when classifying Tritons’ marks.

model	optimizer	activation function	accuracy	loss
ResNet 50	Adagrad	relu	0.9170	0.2357
VGG19	Adagrad	swish	0.8520	0.3967
DenseNet 201	Adagrad	swish	0.8809	0.2958

The stacked model, which combines all three networks, achieved an accuracy of 91.7% when using an extra-randomized tree meta-learner, and 91.6% when using a random forest.

**Figure 2.**
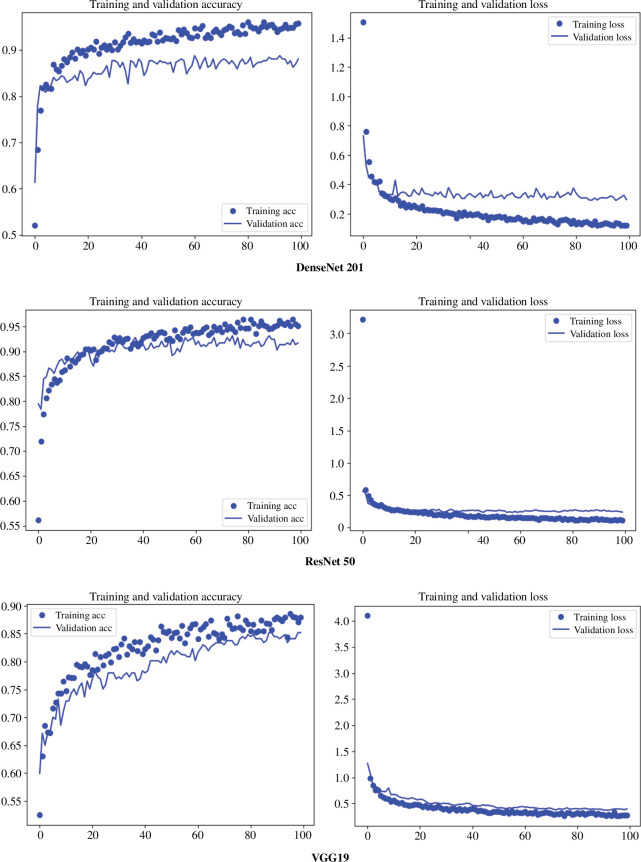
Model accuracy (left) and loss (right) for DenseNet 201, ResNet 50 and VGG19 (top to bottom).

Examining the learning graphs, we can see that the regularization method prevented overfitting, with training and testing errors aligning well. ResNet 50, the top-performing model, exhibits the highest accuracy and the best training fit, while the other networks also demonstrate strong learning processes and good performance (figure 2).

When applied to the Pleistocene sample from Tritons, 34 out of 46 tooth marks (74%) were securely attributed to leopards, as inferred from the coincidence in the classification by the three DL models, which display high probability of accurate classification (table 2). Models differed in the classification of 12 marks; however, in half of that subsample, at least two models coincided in attributing those marks to hyenas. On only one occasion, the models spotted one mark probably made by wolves. Given the potential range of variation of error (spanning from 8% in the ResNet 50 model to 15% in the VGG19 model), we do not feel confident that this individual mark could be effectively interpreted as caused by wolves. Maybe some of the marks attributed by at least two models are not securely identified as hyena-made, but if they were, this would imply a fairly small impact of these predators in the modification of the assemblage. The overwhelming identification of most tooth marks from the Triton assemblage as leopard-made, as supported by the coincidence of the three DL architectures and the resulting high probabilities, emphasize that the main role as accumulator and bone modifier must be attributed to those felids.

**Table 2.** Probability of classification over 1 for each Tritons’ tooth mark according to model.

mark	ResNet 50			VGG19			DenseNet 201		
	**hyena**	**leopard**	**wolf**	**hyena2**	**leopard3**	**wolf4**	**hyena5**	**leopard6**	**wolf7**
**1**	0.0001	0.99	0	0.07	0.91	0.009	0.003	0.99	0
**2**	0	0.99	0	0.03	0.96	0.01	0.001	0.99	0
**3**	0	0.99	0	0.003	0.99	0.001	0	0.99	0
**4**	0	0.99	0	0.05	0.94	0.0006	0	0.99	0
**5**	0	0.99	0	0	0.99	0	0	0.99	0
**6**	0	0.99	0	0.05	0.94	0	0.09	0.87	0.03
**7**	0	0.99	0	0.009	0.98	0	0	0.99	0
**8**	0	0.99	0	0	0.99	0	0	0.99	0
**9**	0.0004	0.99	0.0006	0.02	0.97	0.002	0.03	0.95	0.02
**10**	0	0.99	0.0014	0	0.99	0	0	0.99	0
**11**	0	0.99	0	0.002	0.99	0.002	0	0.99	0
**12**	0.15	0.84	0	0.03	0.96	0.01	0.008	0.97	0.01
**13**	0.07	0.01	0.91	0.69	0.04	0.26	0.97	0.01	0.02
**14**	0	0.99	0	0.008	0.98	0.006	0	0.99	0
**15**	0	0.99	0	0.25	0.54	0.21	0	0.99	0
**16**	0	0.99	0	0.05	0.94	0.01	0	0.99	0
**17**	0	0.99	0	0.002	0.99	0	0	0.99	0
**18**	0	0.99	0.001	0	0.99	0	0	0.99	0
**19**	0	0.99	0	0.01	0.98	0.01	0	0.99	0
**20**	0	0.99	0	0	0.99	0	0	0.99	0
**21**	0	0.99	0	0.29	0.69	0.01	0	0.99	0
**22**	0.005	0.99	0	0.02	0.98	0	0.03	0.96	0.01
**23**	0.05	0.92	0.014	0.1	0.89	0.01	0.13	0.74	0.11
**24**	0	0.99	0	0.003	0.99	0	0	0.99	0
**25**	0	0.99	0	0.03	0.96	0.01	0.01	0.96	0.02
**26**	0	0.99	0	0.54	0.45	0	0.11	0.87	0.007
**27**	0.58	0.41	0.01	0.96	0.03	0.01	0.14	0.85	0
**28**	0.93	0.05	0.02	0.44	0.33	0.21	0.31	0.67	0.02
**29**	0	0.99	0	0	0.99	0	0	0.99	0
**30**	0.001	0.99	0.001	0	0.99	0	0	0.99	0
**31**	0.18	0.03	0.78	0.67	0.31	0.002	0.95	0.04	0.002
**32**	0.76	0.23	0.005	0.32	0.67	0.001	0	0.99	0
**33**	0.009	0.98	0.004	0.94	0.03	0.02	0.03	0.93	0.02
**34**	0.79	0.2	0.001	0.21	0.78	0	0.08	0.91	0.01
**35**	0	0.99	0	0.01	0.68	0.3	0.005	0.98	0.009
**36**	0.003	0.51	0.48	0.13	0.73	0.13	0.16	0.56	0.26
**37**	0	0.99	0	0.58	0.17	0.24	0.002	0.98	0.01
**38**	0	0.99	0.003	0.05	0.92	0.02	0.15	0.98	0
**39**	0	0.99	0	0.023	0.15	0.82	0.01	0.97	0.02
**40**	0.001	0.05	0.94	0.01	0.01	0.98	0.015	0.15	0.83
**41**	0	0.99	0	0	0.99	0	0	0.99	0
**42**	0	0.99	0	0	0.99	0	0	0.99	0
**43**	0	0.99	0	0	0.99	0	0.01	0.98	0.01
**44**	0.01	0.98	0.002	0	0.99	0	0.05	0.89	0.05
**45**	0	0.99	0	0.47	0.13	0.38	0.01	0.94	0.04
**46**	0	0.99	0	0.01	0.98	0.01	0	0.99	0

This is consistent with the initial hypothesis proposed by Micó *et al*. [53], which suggested that leopards were the primary accumulators of the Iberian ibex (*Capra pyrenaica*); whereas the occasional intrusion and secondary activity of other predators, including hominins and/or hyenas, cannot be ruled out.

## 5. Discussion

### 5.1. Summary of the taphonomic evidence for agency in the formation of the Tritons assemblage

The faunal assemblage found at Tritons Cave provides valuable insights into the taphonomic processes and the potential interactions between different carnivores and hominin groups during the Late Pleistocene. The presence of various species and the specific patterns of bone accumulation and modification offer a complex picture of the site formation history, illustrating the challenges that taphonomy must face.

The chronology from Tritons Unit 2 may suggest the potential involvement of multiple carnivores such as leopards (*P. pardus*), bears (*U. arctos* and *U. spelaeus*), wolves (*Canis lupus*), dholes (*Cuon alpinus*)*,* foxes (*V. vulpes*) or hyenas (*Crocuta crocuta spelaea*), all of them present in the area during the Late Pleistocene [54–62].

The current taphonomic behaviour of these predators is relatively well-known, and there is no indication that it significantly differed in the past. For instance, in karstic environments, the signal of bears is primarily associated with their hibernation activities and the consumption of carcasses of their own species that died during this process [3,56,84] No evidence of transport habits has been observed [3], so the presence of ungulate remains in caves frequented by bears is typically attributed to the actions of other predators.

Wolves have traditionally been viewed as significant accumulators of bones in their dens. However, recent observations indicate occasional transport of isolated remains [9,85]. Nevertheless, these studies have not reported the transport of whole carcasses or substantial anatomical portions. Hence, the contribution of wolves to the formation of large bone assemblages must be considered limited, and mostly restricted to a post-depositional ravaging agent. In the Tritons sample, one mark is classified as made by a wolf. Their contribution to the assemblage modification, thus, is marginal.

Few studies have described fox burrows from a taphonomic perspective [4,10]. These animals tend to accumulate significant remains of very small prey, primarily leporids. However, when it comes to larger animals, whether hunted or scavenged, the carcasses are typically quartered, and the different portions, mainly the legs, are hidden in various points near the site of origin (within a range of 50 to 200 m), following a radial dispersion pattern, which it is not the case here. In addition, foxes are commonly not known to accumulate carcasses of the size of the animals represented at Tritons. Therefore, the accumulation observed in the Tritons Cave does not appear to result from the activity of these predators [4].

Owing to their significant impact on bone assemblages, hyenas are among the most extensively studied carnivores, both behaviourally and taphonomically (see [86–93]). However, the faunal assemblage of Tritons Cave does not exhibit the primary features typically found in the dens of these predators. These include the presence of hyena remains, particularly cubs, a wide diversity of ungulates of varying sizes and weights, skeletal profiles primarily dominated by limb bones, a high incidence of toothmarks and fractures and the presence of coprolites. Nevertheless, the models used indicate, with some margin of error (i.e. non-unanimously), that some toothmarks might have been generated by spotted hyenas, suggesting that the occasional presence of these animals in the assemblage cannot be definitively ruled out. For example, mark 13 is classified as hyena by VGG19 and DenseNet 201, but not by ResNet 50. Marks 27–28 are classified as hyena by ResNet 50–VGG19, but not by DenseNet 201.

Leopards, known as territorial predators, are highly effective hunters, particularly targeting small ungulates (>50 kg) on the prowl [94,95]. They often safeguard their prey by either transporting the entire carcass into caves or, if that’s not feasible, by hoisting them up into trees.

The skeletal profile of the faunal assemblage in Tritons Cave, characterized by an abundance of axial elements and limbs with epiphyses [53], is consistent with leopard accumulations described in other sites [60,61] The presence of tooth marks predominantly on the proximal epiphyses of long bones further supports the likelihood of leopard involvement [11].

The lion (*P. leo spelaea*), another felid species present in the Iberian Peninsula, is not usually linked to bone accumulations because of its behaviour of consuming prey at the kill site [84], and tending to focus on larger prey than the Iberian ibex [64]. In addition, if taking similarly sized carcasses eaten by lions as proxies, their damage on these smaller animals is much more intensive than documented in the Tritons ibex sample [68], which further supports discarding lions as potential agents contributing to the Tritons faunal assemblage.

The evidence is scarce regarding human involvement, with only a small number of flakes and bones bearing cut marks. These artefacts suggest sporadic human presence in the natural shelter, but their limited number makes it challenging to draw definitive conclusions about the extent of human interactions with the carnivores and their impact on the recovered faunal assemblage [53].

While all these data contribute to confirming the hypothesis developed by Micó *et al*. [53] based on traditional taphonomy, our models suggest a greater complexity in the formation of the assemblage. Therefore, Unit 2 must be considered a palimpsest, where the primary regular dynamic (alternation between refuges of leopards and hibernation places for bears) was occasionally disrupted by the presence of other predators (such as hyenas and probably wolves). The nature of these occasional carnivores as either accumulators or merely scavengers, as well as their intensity, remains unknown. However, it is sufficient to suggest a diversified environment in a region very close to the Central Axial Pyrenees, dominated during the MIS 2 by glacial and periglacial climates.

In summary, the data obtained from the taphonomic analysis of Unit 2 from Tritons Cave provides valuable information about the complex interactions between different carnivores and hominin groups during the Late Pleistocene. The primary agent responsible for the accumulation of the Iberian ibex appears to be the leopard, while other carnivores, such as brown bears, wolves or hyenas, may have impacted the formation of the assemblage to a lesser extent, considering the evidence of peeling and/or digestion. However, this overlap of processes can raise doubts in several cases about primary and secondary agents. This is why we applied a more objective analytical method based on CV and DL.

### 5.2. Contribution of artificial intelligence to the taphonomic interpretation of Tritons

AI, particularly through the application of DL and CV, holds significant potential for advancing taphonomic studies, especially in the analysis of BSM caused by carnivores. Traditionally, the identification and interpretation of BSM have relied heavily on the expertise and subjective judgement of researchers. This method, while effective, is prone to biases and inconsistencies, which can lead to misinterpretations. The advent of AI offers a promising solution to these challenges by providing more objective, consistent and scalable methods for analysing BSM. AI methods, particularly DL and CV, have shown remarkable success in image analysis tasks across various fields. In taphonomy, these technologies can be used to analyse high-resolution images of bones, identifying and classifying BSM with a level of precision and reliability unattainable by traditional methods. DL involves the use of neural networks with many layers (deep neural networks) to model complex patterns in data. In the context of BSM analysis, these models can be trained on large datasets of labelled bone images, learning to recognize subtle differences between marks made by different agents: several studies have demonstrated how DL and CV can be employed to distinguish between marks made by different carnivores [44–46,75]. For example, AI can differentiate between tooth marks left by different species of predators, such as lions, hyenas and leopards, based on specific microscopic and morphological characteristics of the tooth marks [75]. AI enables the quantitative analysis of BSM, providing metrics that can be statistically analysed. This approach enhances the objectivity of taphonomic studies and allows for more robust comparisons between different sites.

As we have shown here, AI should complement, not replace, traditional taphonomic methods. Integrating AI with expert knowledge and manual analysis can enhance the overall reliability and depth of taphonomic studies. Given that traditional taphonomic methods supported an interpretation of leopards as the primary accumulating (and bone modifying) agent, we tested this interpretation here.

When applying AI to the Tritons faunal assemblage, a comprehensive study evaluating three DL architectures—ResNet 50, VGG19 and DenseNet 201—for classifying BSM, yielded models which achieved accuracy estimates exceeding 85% on the experimental testing dataset. Among them, ResNet 50 emerged as the most effective, attaining nearly 92% accuracy. DenseNet 201 and VGG19 followed closely with accuracies of 88% and 85%, respectively, though with slightly higher loss values.

A stacked model combining all three networks was also assessed, reaching an accuracy of 91.7% with an extra-randomized tree meta-learner and 91.6% with a random forest. The learning graphs indicated effective regularization across models, preventing overfitting and aligning training and testing errors. ResNet 50 not only exhibited the highest accuracy but also demonstrated the best training fit, while DenseNet 201 and VGG19 also showed robust learning and good performance. These highly accurate models on the experimental dataset constitute a solid referential framework, and provides confidence in the classification of tooth marks from prehistoric faunal assemblages when applied to them.

Applying these models to a Pleistocene sample from Tritons, the study found that 34 out of 46 tooth marks (74%) were confidently attributed to leopards, as evidenced by consistent classification across all three DL models. There was some discrepancy in the classification of 12 marks, with at least two models attributing half of these to hyenas. Only one mark was tentatively identified as possibly wolf-made, but the confidence in this classification was low owing to error ranges from 8% (ResNet 50) to 15% (VGG19). This small subsample of indeterminate marks raises the issue of potential ways to improve the models’ performance in the future by increasing the quality of the images. For example, higher resolution methods such as reflectance transformation imaging, could aid CNNs in better discriminating the shape of tooth marks produced by different carnivores.

These results show that: (i) the experimental assemblage yielded models with high accuracy, and thus, high reliability in mark classification; (ii) when running the 46 tooth marks from the Tritons assemblage through these models, most of them were classified as made by leopard with high probability (table 2); and (iii) only a few marks were shown to have potential modifications introduced by hyenas, which would show some minor role in the post-depositional history of the assemblage and discards these carnivores as the primary agents in the accumulation.

This overwhelming identification of tooth marks as leopard-made, supported by high classification probabilities across the DL architectures, suggests that leopards were the primary accumulators and bone modifiers in the Triton assemblage. This finding aligns with the hypothesis by Micó *et al*. [53] that leopards primarily accumulated Iberian ibex (*Capra pyrenaica*) remains, although secondary activities by other predators like hominins and hyenas cannot be entirely ruled out.

The present study emphasizes the high accuracy of DL methods on experimentally controlled samples and their potential for usefully differentiating among carnivore taxa in prehistoric assemblages. The role of machine learning, and more specifically CV, in taphonomic research is only in its initial stages, but it has a bright future ahead. The major downside to DL methods is that they require large datasets for training. To counter this problem, alternative methods on smaller datasets are catching up in their accuracy and reliability. N-way-k-shot meta-learning methods are yielding good results on smaller samples, but they have never been applied to taphonomic problems before. Although they are good solutions for small samples, they still are far from the generalization qualities of DL methods. This wide array of techniques will open new windows of agency detection to taphonomic research and to other areas of palaeobiology in the near future.

## 6. Conclusions

While traditional taphonomic methods are generally effective in distinguishing various carnivoran agents, there are instances where significant overlap exists, leading to uncertainty regarding the primary and secondary agents involved. Factors such as equifinality and other preservation-related processes can contribute to the modification and deletion of tooth marks and skeletal elements, highly complicating the identification process [12]. Furthermore, the great majority of techniques usually applied to study BSMs rely on highly subjective variables, so the results and conclusion may defer greatly depending on the expert and the way the raw data is treated [96].

Here, we analysed a sample of 45 well-preserved two-dimensional images of tooth marks from Tritons Cave and compared them with experimental tooth marks modelled in three different neural network models. The goal was to determine the processes and agents that contributed to the formation of Tritons Unit 2. The authors selected the clearest and best-preserved tooth marks from the original study to avoid possible biases, and tested diverse DL architectures, optimizers and functions to achieve the most accurate result.

The present study illustrates that the use of appropriate tools, such as neural networks, enables the specific identification of carnivores and the achievement of high precision in the classification of bone surface modifications. These promising findings provide a more objective approach to identifying taphonomic processes, facilitating faster and more efficient assessments of agency in the formation and alteration of archaeological assemblages. Furthermore, the recognition of leopards as the primary accumulators at the studied site offers valuable insights into these types of cave deposits. It also establishes criteria for distinguishing leopard accumulations from those created by other carnivores and provides guidelines for their identification in anthropic contexts.

While previous DL studies have predominantly concentrated on experimental datasets, this paper showcases its potential to advance our comprehension of actual archaeological and palaeontological contexts. These methods enable us to deduce patterns and trends, achieving more comprehensive classifications and higher resolutions. This, in turn, leads to the revelation of valuable behavioural insights that significantly enhance the understanding of our past. The findings from this study hold the potential to make significant contributions to the broader field of taphonomy, opening new avenues for future research in the identification of carnivore agency and the extraction of meaningful insights from archaeological and palaeontological records.

## Data Availability

Data are accessible publicly in Harvard Dataverse: [97,98]. Supplementary material is available online [99].
